# Hierarchizing caries risk factors among first-year university students in Nice (France): a cross-sectional study

**DOI:** 10.1186/s12903-017-0452-8

**Published:** 2017-12-21

**Authors:** Romain Ceinos, Marie-France Bertrand, Céline Cucchi, Laurence Lupi

**Affiliations:** 1 Université Côte d’Azur, UFR Odontologie, 24 avenue des diables bleus, 06357 Nice cedex 4, France; 2 Université Aix-Marseille, ADES, bd Pierre Dramard, 13334, Marseille cedex 15, France; 3 Université Côte d’Azur, MICORALIS, 24 avenue des diables bleus, 06357 Nice cedex 4, France; 4 Centre Hospitalier Universitaire de Nice, Pôle d’Odontologie, 5 Rue Dévoluy, 06000 Nice, France; 5 UFR Odontologie, Pôle Universitaire Saint Jean d’Angély, 24 avenue des diables bleus, 06357 Nice cedex 4, France

**Keywords:** Health-related behavior, Dental caries, First-year university students, Multivariate analysis

## Abstract

**Background:**

The purpose of this study was to rank the risk factors for dental caries among first-year university students in Nice (France).

**Methods:**

All first-year students are required to undergo a compulsory preventive medical examination. Among these students, volunteers were offered a dental visit. Information was collected through an interview followed by an oral examination. We assessed the volunteers’ oral hygiene habits (daily toothbrushing frequency, type of toothbrush used, frequency of toothbrush replacement, place of toothpaste purchase, and flossing), daily health-related behaviors (number of main daily meals, daily sugary intakes, smoking, alcohol consumption, consumption of cannabis or other drugs), oral-health-related behaviors (self-reported oral health, dental visits during the past year, reason for the last dental consultation, and failure to seek dental care due to financial reasons), and oral health issues (dental crowding, oral hygiene, presence of caries, presence of pit and fissure sealant remnants). The dependent variable was the presence of at least one untreated carious lesion. The data were subjected to univariate analyses to select explanatory variables, and subsequently, a logistic regression was performed.

**Results:**

Six hundred twenty-nine students aged 18.8±1.6 years were enrolled in this study. The sex ratio was 0.72, with a strong predominance of the female gender. Only 59.3% of the students had never experienced dental caries, while 22.4% had already undergone restorative procedures and did not have any carious lesion at the time of the examination, and 11.6% presented with carious lesions and had never been treated by a dentist. Lastly, 6.7% had carious lesions despite evidence of prior restorative procedures. The multivariate analysis revealed the following pejorative risk factors: failure to seek dental care due to financial reasons (OR:3.06, 95% CI: 1.40–6.70), poor oral hygiene revealed during the oral examination (OR:2.59, 95% CI: 1.60–4.20), and poor self-reported oral health (OR:2.43, 95% CI: 1.24–4.77). Conversely, the analysis revealed the following protective factors: preventive visits to the dentist (OR:0.63, 95% CI: 0.41–0.99), the use of an electric toothbrush (OR:0.36, 95% CI: 0.17–0.77), and sealant remnants (OR:0.22, 95% CI: 0.05–0.97).

**Conclusions:**

The highest-ranking caries risk factor for the study population was the financial barrier.

**Electronic supplementary material:**

The online version of this article (10.1186/s12903-017-0452-8) contains supplementary material, which is available to authorized users.

## Background

In 2006, a French national report revealed that university students neglected their health [1]. These young adults think they are healthy and feel invulnerable. In general, they had poor dietary and oral hygiene habits and often failed to seek dental care. The report also noted risk behaviors, particularly smoking, excessive alcohol consumption, and the daily use of certain drugs considered to be less dangerous than tobacco, particularly cannabis. Over the last 10 years, particular emphasis has been placed upon promoting a balanced diet and the prevention of risk behaviors in French universities.

In a review published in *The Lancet* by Selwitz et al. in 2007, caries risk assessment included physical, biological, environmental, behavioral, and lifestyle-related factors [2]. The individual caries risk can vary with time since many risk factors are changeable. Caries is related to one’s lifestyle, and behavioral factors under a person’s control are clearly implicated [3–6]. These factors include poor oral hygiene [7–9] and poor dietary habits [10–13]. Other factors related to caries risk include socio-economic status [3, 5, 6], the use of dental sealants [14, 15], and dental crowding, which promotes plaque retention and increases the difficulty of maintaining good oral hygiene [16]. Besides, the relative caries preventive effects of fluoride toothpastes of different concentrations increase with higher fluoride concentration [17]. Though, in France, toothpastes sold in pharmacies need a special authorization (AMM) because the amount of fluorides is high (1500 ppm and more), and they are therefore considered as medicines. Because they are less fluoridated (1450 ppm and less), toothpastes in supermarkets are considered as hygiene products.

Numerous studies performed among undergraduate students have concerned dental students [18] but have less often studied the general university student population. However, dental students constitute a selected minority that is clearly demarcated from students of other disciplines regarding a higher awareness of self-reliant oral hygiene [18]. The DMFT score of undergraduate students has been the object of studies in various universities worldwide. Studies in Europe, North America, Asia and the Middle East show that the DMFT score of undergraduate students range from 4.1 ± 3.1 at Sana’a University in Yemen [19] to 3.9 ± 3.9 at San Luis Potosí University in Mexico [20], 2.9 ± 3.3 at Helsinki University in Finland [21], 2.0 ± 2.9 at Okayama University in Japan [22], and 1.2 ± 2.0 at Nice Sophia Antipolis University in France [23].

Low sugar consumption and good oral hygiene are critical for the prevention of caries and the maintenance of good oral health [24]. In Lebanon, 65% of undergraduate students reported brushing their teeth at least twice per day [25], while that percentage was 68% in Turkey [26], 74% in the USA [27], 87% in Japan [28], and 92% in Italy [29] and France [23]. Of subjects in Turkey, 91% reported never flossing versus 87% in Japan, 80% in France, 72% in Lebanon, 35% in Italy and 20% in the USA. Of the students in France and Italy, 62% and 60%, respectively, reported visiting a dentist during the previous year, while these percentages were only 31% in Lebanon and 30% in Turkey. High sugar consumption has long been linked to dental caries [30]. A quarter of USA Midwestern university students reported eating sugary foods (including foods and drinks, such as candies, soft drinks, juices, doughnuts, jellies, etc.) more than once daily. Among the 89% of students who reported drinking pop, 69% selected regular pop as their favorite as opposed to diet pop [27].

Smoking habits may also be associated with a higher risk of dental caries [31]. Self-reported use of tobacco among adolescents was significantly associated with an increased incidence of dental caries over a 3-year period [32]. This association was also apparent with alcohol [33–35] and drug abuse [36–38].

No data are available in France concerning the specific caries risk factors among university students. However, in France, university students often leave their family for the first time to study at a university that occasionally is far from home [39]. This assertion of autonomy may be particularly risky for their oral health. A national survey was conducted in Finland, and based on the university health department registries, an electronic questionnaire was sent to first-year students on their general, emotional, and oral health [40]. This survey revealed an increased risk of deterioration of oral health and thus a need for treatment in relation to poor habits regarding oral health and diet.

In France, all university students in their first 3 years of study are required to attend a preventive medical consultation [41]. In the Côte d’Azur University, this consultation takes place during the first year of study. A preventive dental consultation conducted by final-year dental students has also been implemented for more than 10 years. The purpose of this study was to rank the risk factors for dental caries among first-year university students.

## Methods

### Study population

A cross-sectional epidemiological study was conducted at the Côte d’Azur University. The study was approved by the University institutional review board (Commission de la Formation et de la Vie Universitaire). A previous study conducted on 4929 students of Nice-Sophia Antipolis University from 2009 to 2011 revealed that 43% of the students had at least one carious lesion [23]. The sample size calculation provided a number of at least 377 subjects for an absolute error of 5% and a type I error of 5%.

### Investigators calibration

The investigators were ten final-year dental students who were previously trained to work in pairs. The questionnaire with closed questions was developed, and its use according to standardized modalities was validated for previous studies (Additional file 1) [23, 42, 43]. A very descriptive document was used to explain all of the recorded variables and the manner in which they should be coded. Sessions of tutorial classes were conducted by an experienced teacher to calibrate the operators to ensure that the interviews were led according to standardized modalities.

Our final-year dental students have a clinical experience of 2 years. Sessions of tutorial classes were also conducted to calibrate the operators. During these sessions, the inter- and intra-operator reliabilities (at a one-week interval) were assessed. Each operator was trained and became eligible for the study if all of the calculated Cohen kappa values (for qualitative variables) and intra-class correlation coefficients (for quantitative variables) exhibited minimum acceptable inter- and intra-operator agreement of more than 0.80.

### Recruitment procedure

Participation in the study was proposed to all first-year undergraduate students who attended the medical examination in the first half of 2015. All students were eligible for the study with no exclusion criteria. After information on the objectives of the study was provided and the student’s consent was obtained, information was collected through an interview and an oral examination. The interview and oral examination were immediately conducted in the unit for medical examination in the university department of preventive medicine, which was adapted for this purpose.

### Collected data

A closed room was equipped with a dentist chair to provide privacy for study participants. Appropriate dental lighting was a part of the unit. Disposable periodontal examination kits with a probe, mirror and cotton rolls were used, as were masks and disposable gloves. Data entry was anonymous, and no information in the electronic file could be used to identify the source person. The students were interviewed using standardized modalities and a questionnaire with closed questions about sociodemographic data that included the following: areas of study (Health and Sport, Science and Technology, Law and Economics, Management and Business, or Letters and Arts and Humanities), age, gender, social security coverage (French social security system, health mutual or supplementary universal health-care coverage, or other systems), father’s and mother’s professions (management profession, employees, or without employment), accommodation status (living with their parents or away from the parental home for at least part of the time), part-time jobs, diet-related behaviors (number of main daily meals; number of snacks between meals; daily consumption of sugary drinks, candies, sugar-free chewing gums; the drink consumed when thirsty, i.e., water or other drinks), oral hygiene habits (daily toothbrushing frequency, i.e., one, two or three times or not every day), type of toothbrush used (electric or manual), frequency of toothbrush replacement (at least every 3 months), place of toothpaste purchase (pharmacy or supermarket), flossing, addictive behaviors (smoking, alcohol consumption, consumption of cannabis or other drugs), oral health-related behaviors (self-reported oral health, i.e., good or poor health; dental visits during the past year; reason for the last dental consultation, i.e., preventive or curative; and failure to seek dental care due to financial reasons).

The oral examination assessed dental crowding by measuring the most crowded arch (no crowding between 0 and 2 mm, mild or moderate crowding between 2.1 and 7.0 mm, and severe crowding more than 7 mm) [44], periodontal status using the Community Periodontal Index of Treatment Needs (CPITN) (0: no signs of disease; 1: gingival bleeding after gentle probing; 2: presence of supra or subgingival calculus; 3: shallow pockets of 4–5 mm; and 4: pockets of 6 mm or more), oral hygiene using the Silness-Löe plaque index (0: no observable plaque; 1: a thin film of plaque detected at the gingival margin by running a probe across the tooth surfaces; 2: a moderate amount of plaque detected along the gingival margin with plaque visible clinically; and 3: heavy plaque accumulation detected at the gingival margin and in the interdental spaces), the calculus index (0: no observable calculus; 1: scattered calculus covering less than one-third of the buccal surface of the tooth; 2: calculus covering between one- and two-thirds of the buccal tooth surface with minimal subgingival calculus; and 3: calculus covering greater than two-thirds of the buccal tooth surface and extending subgingivally), the presence or absence of pit and fissure sealant remnants, the presence or absence of restorative care, and the DMFT index (decayed, missing, or filled teeth; only cavitated carious lesions were considered in this screening situation).

### Statistical analysis

The risk factors were initially subjected to univariate analyses. The dependent variable was dichotomous (i.e., the presence or absence of at least one carious lesion at the time of the oral examination). The independent variables with more than two categories were dichotomized as follows for the statistical analyses: Health/Sport area of study vs others, father’s profession in management vs others, mother’s profession in management vs others, at least 3 main meals per day vs less than 3 main meals per day, at most one snack per day vs more than one snack per day, no daily soft drink consumption vs daily consumption, toothbrushing at least twice per day vs less than twice per day, daily alcohol consumption vs no daily consumption, and preventive reason for the last dental consultation vs curative or unknown reason. To determine which variables were significantly related to our variable of interest, each variable was examined separately from the others, and the chi-square test (or Fisher’s exact test) was applied. The *p* value was set at 0.05.

A multivariate logistic regression analysis was used to identify the risk factors that predicted the development of carious lesions. Any independent variable that yielded a significant univariate test result at the level of 0.25 was selected as a candidate for the multivariate analysis. The major purpose of this technique was to quantify the strengths of the associations between the dependent variable and each of the independent variables (odds ratios) by accounting for the effects of the other model-integrated variables. The main advantage of this approach is the avoidance of confounding effects caused by analyzing the associations of all variables together. The forward stepwise likelihood ratio method for analyses was used.

## Results

Six hundred twenty-nine students in their first year of study were enrolled in this study (Table 1). The average age was 18.8 ± 1.6 years. The sex ratio was 0.72, with a strong predominance of the female gender. The DMFT was 1.20 ± 2.07 (D = 0.43 ± 1.20; M = 0.02 ± 0.13; and F = 0.75 ± 1.63). Only 59.3% of the students had never experienced dental caries, while 22.4% had already undergone restorative procedures and did not have any carious lesions at the time of the examination, and 11.6% presented carious lesions and had never been treated by a dentist (Table 2). Lastly, 6.7% had carious lesions despite evidence of prior restorative procedures.

**Table 1:** Background factors, daily health-related behaviors and oral-health behaviors among the first-year students of Nice Côte d’Azur University

	N	%
Gender		
Male	263	41.8
Female	366	58.2
Area of study		
Health, Sport	275	43.7
Science, Technology	91	14.5
Law, Economics, Management, Business	163	25.9
Letters, Arts and Humanities	100	15.9
Social security coverage		
French social security system	36	5.7
French social security system + health mutual or supplementary universal health-care coverage	588	93.5
Other systems	5	0.8
Father’s profession		
Managing profession	313	49.8
Employees	214	34.0
Without employment	70	11.1
Not documented or deceased	32	5.1
Mother’s profession		
Managing profession	202	32.1
Employees	301	47.9
Without employment	116	18.4
Not documented or deceased	10	1.6
Accommodation		
Living with their parents	345	54.8
Away from parental home at least a part of the time	284	45.2
Part-time job		
Yes	86	13.7
No	543	86.3
Number of main daily meals		
One meal	12	1.9
Two meals	167	26.6
Three meals	446	70.9
Four meals or more	4	0.6
Number of snacks between meals		
None	142	22.6
One snack	368	58.5
Two snacks or more	119	18.9
Daily consumption of sugar drinks		
None	298	47.4
One drink	251	39.9
Two drinks or more	80	12.7
Daily consumption of candies		
Yes	74	11.8
No	555	88.2
Daily consumption of sugar-free chewing gums		
Yes	133	21.1
No	496	78.9
Drink consumed when thirsty		
Water	483	76.8
Other drink	146	23.2
Daily toothbrushing frequency		
One time	78	12.4
Two times	446	70.9
Three times	101	16.1
Not every day	4	0.6
Type of toothbrush used		
Manual	530	84.3
Electric	99	15.7
Toothbrush replacement at least every three months		
Yes	460	73.1
No	169	26.9
Place of purchase of toothpaste		
Pharmacy	58	9.2
Supermarket	490	77.9
Not known	81	12.9
Flossing		
Yes	76	12.1
No	553	87.9
Smoking		
Yes	162	25.8
No	467	74.2
Cannabis consumption		
Yes	75	11.9
No	554	88.1
Other drugs consumption		
Yes	17	2.7
No	612	97.3
Alcohol consumption		
Never	226	35.9
Occasional	388	61.7
Daily	15	2.4
Self-reported oral health		
Good health	580	92.2
Poor health	49	7.8
Dental visit during the past year		
Yes	369	58.7
No	260	41.3
Reason for the last dental consultation		
Preventive	431	68.5
Curative	167	26.6
Not known	31	4.9
Failing to seek dental care due to financial reasons		
Yes	34	5.4
No	595	94.6

**Table 2:** Oral examinations among first-year students of Nice Côte d’Azur University

	N	%
Dental crowding		
No crowding	501	79.7
Mild or moderate crowding	102	16.2
Severe crowding	26	4.1
Periodontal status		
No signs of disease	482	76.6
Gingival bleeding	66	10.5
Supra- or subgingival calculus	80	12.7
Shallow pockets	1	0.2
Poor oral hygiene (plaque and/or calculus)		
No	507	80.6
Yes	122	19.4
Presence of at least one carious lesion		
No	514	81.7
Yes	115	18.3
Presence of restorative care		
No	446	70.9
Yes	183	29.1
Pit and fissure sealant remnants		
No	591	94.0
Yes	38	6.0

### Results of the univariate analyses and selection of the explanatory variables

The main variables associated with the presence of at least one carious lesion were the professional status of the mother, i.e., whether she had an intermediate professional position as opposed to being unemployed or having a higher professional position (*p* = 0.028); the use of an electric toothbrush (*p* = 0.010); the presence of sealant remnants (*p* = 0.030); a preventive visit to the dentist (*p* = 0.001); smoking (*p* = 0.033); drinking something other than water when thirsty during the day (p = 0.028); periodontal disease, even mild cases, revealed during the oral examination (*p* = 0.020); poor oral hygiene revealed by presence of dental plaque and/or calculus (*p* < 0.001); self-reported poor oral health (p < 0.001); and failing to seek care due to financial reasons (p < 0.001) (Table 3).

**Table 3:** Selection of the explanatory variables for inclusion in the logistic regression model (*p* < 0.25)

		At least one active carious lesion (n)		Univariate Analysis
		NO	YES	P
Gender	Female	297	69	0.677
	Male	217	46	
Area of study	Health, Sport	222	53	0.604
	Others	292	62	
Father’s profession	Managing profession	264	49	0.099
	Others	250	66	
Mother’s profession	Managing profession	175	27	0.028^a^
	Others	339	88	
Accommodation	Living with their parents	285	60	0.536
	Others	229	55	
Part-time job	Yes	64	22	0.071
	No	450	93	
At least 3 main meals per day	Yes	375	75	0.109
	No	139	40	
At the most one snack per day	Yes	414	96	0.513
	No	100	19	
No daily soft drink consumption	Yes	253	45	0.063
	No	261	70	
Water consumption when thirsty	Yes	404	79	0.028^a^
	No	110	36	
Daily candy consumption	Yes	55	19	0.108
	No	459	96	
Daily sugar-free chewing gum consumption	Yes	102	31	0.101
	No	412	84	
Toothbrushing at least twice per day	Yes	447	100	1.000
	No	67	15	
Use of electric toothbrush	Yes	90	9	0.010^a^
	No	424	106	
Flossing	Yes	68	8	0.080
	No	446	107	
Toothbrush replacement at least every 3 months	Yes	380	80	0.353
	No	134	35	
Purchase of toothpaste at the pharmacy	Yes	49	9	0.721
	No	465	106	
Daily smoking	Yes	123	39	0.033^a^
	No	391	76	
Cannabis consumption	Yes	60	15	0.637
	No	454	100	
Another drugs consumption	Yes	12	5	0.214
	No	502	110	
Daily alcohol consumption	Yes	12	3	0.740
	No	502	112	
Presence of dental crowding	Yes	99	29	0.160
	No	415	86	
Periodontal disease, even mild	Yes	110	37	0.020^a^
	No	404	78	
Sealant remnants	Yes	36	2	0.030^a^
	No	478	113	
Presence of restorative care	Yes	141	42	0.055
	No	373	73	
Poor oral hygiene (plaque and/or calculus)	Yes	82	40	< 0.001^a^
	No	432	75	
Dental visit during the past year	Yes	305	64	0.466
	No	209	51	
Reason for last dental consultation	Preventive	367	64	0.001^a^
	Other reason	147	51	
Self-reported oral health	Good	486	94	< 0.001^a^
	Bad	28	21	
Failing to seek dental care due to financial reasons	Yes	19	15	< 0.001^a^
	No	495	100	

^a^Significant

### Ranking of the risk factors

A regression analyses model was designed with the possible explanatory factors from the socioeconomic data (father’s profession, mother’s profession, and part-time job), dietary habits (at least 3 main meals per day, no daily soft drink consumption, water consumption when thirsty, daily candy consumption, and daily sugar-free chewing gum consumption), oral hygiene habits (use of an electric toothbrush and flossing), addictive behaviors (daily smoking and drug consumption), oral health-related behaviors (reason for last dental consultation, self-reported oral health, and failure to seek dental care due to financial reasons) and clinical data (presence of dental crowding, periodontal disease (even mild), sealant remnants, presence of restorative care, and poor oral hygiene revealed by presence of dental plaque and/or calculus).

The multivariate analysis identified pejorative risk factors and ranked them as follows: failure to seek dental care due to financial reasons (OR:3.06, 95% CI: 1.40–6.70), poor oral hygiene revealed during the oral examination (OR: 2.59, 95% CI: 1.60–4.20), and poor self-reported oral health (OR: 2.43, 95% CI: 1.24–4.77) (Table 4). Conversely, this study also highlighted and ranked the protective factors as follows: a preventive visit to the dentist (OR: 0.63, 95% CI: 0.41–0.99), the use of an electric toothbrush (OR: 0.36, 95% CI: 0.17–0.77), and the presence of pit and fissure sealant remnants (OR: 0.22, 95% CI: 0.05–0.97).

**Table 4:** Results from the logistic regression analyses with the dependent variable “presence of at least one carious lesion”

Variables in the final equation	Sig.	Odds Ratio	95% CI	
			Lower	Upper
Use of an electric toothbrush	0.008	0.360	0.169	0.766
Last dental consultation for a preventive reason	0.045	0.634	0.406	0.990
Presence of sealant remnants	0.045	0.223	0.052	0.969
Poor oral hygiene	0.000	2.585	1.590	4.202
Failing to seek dental care due to financial reasons	0.005	3.060	1.397	6.700
Poor self-reported oral health	0.010	2.428	1.236	4.767

In the final equation, six independent variables remained and were ranked whereas fourteen were excluded: mother’s profession, father’s profession, part-time job, at least 3 main meals per day, no daily soft drink consumption, water consumption when thirsty, daily candy consumption, daily sugar free chewing gum consumption, daily smoking, drug consumption, flossing, presence of dental crowding, periodontal disease even mild, presence of restorative care

## Discussion

The multivariate analysis made it possible to rank the risk factors for dental caries among the first-year university students of Nice in the first half of 2015. These risk factors were failure to seek dental care due to financial reasons followed by poor oral hygiene revealed during the oral examination and poor self-reported oral health.

In 2014, the Student Life Observatory published the results of a national survey about the living conditions of students in France [45]. Among other findings, this survey revealed that 11% of the students were not satisfied with their current health status and that 13% confessed to having already failed to seek medical care due to financial reasons. Many social assistance programs exist, for example, universal supplementary health coverage (CMUc [couverture maladie complémentaire universelle]) (free supplementary health coverage meant to facilitate access to healthcare for the low-income population with a stable and regular residence in France) and payment assistance for the supplementary health aid system (ACS [aide au paiement d’une complémentaire santé]) (aid that offers discounts on the premiums for supplementary health coverage for low-income populations with incomes slightly higher than the limit set for the allocation of CMUc). These programs make it possible to provide 100% dental coverage in France [46]. In 2015, a French national report revealed that 19.1% of students lived below the poverty line, i.e., less than 987 euros per month. The advance of dental fees can be perceived as unaffordable in this context [47].

The presence of dental plaque and/or calculus revealed during the oral examination can likely be more strongly attributed to the inadequate behavior of some students. Nevertheless, the French Union for Oral Health (UFSBD [Union Française pour la Santé Bucco-Dentaire]) issues good practice guidelines for the general public to ensure adequate oral hygiene. More specifically, this organization focuses on adults under the age of 40 and recommends at least two brushings per day, the use of fluoride toothpastes, the use of floss in the evening, a preventive visit to the dentist at least once per year, and the use of sugar-free chewing gums after each food intake [48].

The univariate analysis also highlighted the possible role of periodontal disease, even mild cases. Yet, gingivitis is often correlated with the presence of plaque, which is the consequence of a lack of brushing. Both periodontal status and poor oral hygiene were included in the multivariate logistic regression model, and only poor oral hygiene remained a proven risk factor. The main advantage of the multivariate analysis was thus to avoid confounding effects that arise by analyzing the associations of all variables together. Good practice guidelines are not sufficient to enhance a good oral hygiene for students. Actually, in France, there is no dental hygienist to educate patients in regard to diet and oral hygiene habits. Nevertheless, the success of the dental hygienist in the dental health education of patients has long been recognized in terms of influencing behavior [49]. A Swedish study revealed that patients’ attitudes were less negative towards dental hygienists than towards dentists [50]. This difference was more pronounced among students than general patients and patients with periodontal disease. Therefore, this specific population could greatly benefit from the introduction of dental hygienists in France.

The highest-ranking caries protective factor among the study population was the use of dental sealants, followed by the use of an electric toothbrush and a preventive visit to the dentist. Sealants are used and have been recommended in France since the early 2000s, and their efficacy is well documented [51]. The role played by the presence of sealant remnants is thus not surprising. Our results also confirmed recent studies that tend to prove the superiority of the use of electric brushing over manual brushing [52]. Preventive dental consultations remain rare in France despite institutional incentives. Since 2007 the “M’T dents” (love your teeth!) program has been offering all children aged 6, 9, 12, 15 and 18 one free dental consultation and treatment, if necessary [53]. The purpose of this initiative was to develop the habit of preventively visiting the dentist. However, 41% of the students had not visited the dentist in the previous year. Moreover, the reason for the last dental appointment was preventive in only 69% of the cases.

The strengths of our study lie in supplying French data on a rarely studied population. The originality comes from the peer-led action among students. A study conducted in India focused primarily on dental students and revealed the positive attitude of this category of students towards their own oral health [54]. The authors mentioned the potential interest of a peer-driven prevention approach (dental students versus the students of other fields) to promote oral health on university campuses.

The limitations of our study arise from both the participation of only volunteer students and the cross-sectional design, which only provides a snapshot of our outcome at a specific point in time and provides no indication of the sequence of events. The oral examinations were performed in screening conditions, and an underestimation of the number of students affected by caries is possible. However, the use of recognized indices to estimate the dental caries status (i.e., the DMFT index), the oral hygiene (i.e., the plaque index and the calculus index) and the periodontal status (i.e., CPITN) likely limit these biases.

## Conclusions

Students are known to neglect their oral hygiene and are particularly vulnerable to dental decay. The highest-ranking caries risk factor for the study population was the financial barrier.

## Additional file

Additional file 1: questionnaire used for the interview of the students. The questionnaire consisted in five parts including socio-economic information, daily diet-related behaviors, addictive behaviors, oral hygiene habits and oral health–related behaviors. (DOCX 17 kb)

## Abbreviations

CI: Confidence interval
CPITN: Community periodontal index of treatment needs
DMFT: Decayed, missing or filled teeth
mm: millimeters
OR: Odd ratio
Sig.: Significance
