# Supplementary material for: Hierarchizing caries risk factors among first-year university students in Nice (France): a cross-sectional study
Source: BMC Oral Health. 2017 Dec 21;17:159. doi: 10.1186/s12903-017-0452-8 (PMC5740936; doi:10.1186/s12903-017-0452-8)
Supplement: Additional file 1: — questionnaire used for the interview of the students. The questionnaire consisted in five parts including socio-economic information, daily diet-related behaviors, addictive behaviors, oral hygiene habits and oral health–related behaviors. (DOCX 17 kb) [file 12903_2017_452_MOESM1_ESM.docx]

| **Questionnaire for interview (English version)**  **Age:** ________________ **Gender:** ________________ |
| --- |

**Part A: Sociodemographic data**

1. What is your course of study?

□ Health and Sport

□ Science and Technology

□ Law and Economics

□ Management and Business

□ Letters, Art and Humanities

2. What is your social security coverage?

□ French social security system

□ French social security system + Supplementary health-care coverage

□ French social security system + Supplementary universal health-care coverage

□ Other system__________________

3. What is your father’s profession?

□ Manager

□ Employee

□ Unemployed

□ Deceased

□ Unspecified

4. What is your mother’s profession?

□ Manager

□ Employee

□ Unemployed

□ Deceased

□ Unspecified

5. Where are you living?

□ Living with your parents

□ Living away from parental home at least a part of the week

6. Do you have a part-time job

□ Yes

□ No

**Part B: Diet-related behaviors**

1. How many main meals do you eat a day?

□ One meal

□ Two meals

□ Three meals

□ Four meals or more

2. How often do you eat snacks a day?

□ None

□ One snack

□ Two snacks or more

3. Do you daily consume soft drinks?

□ No

□ One soft drink

□ Two soft drinks or more

4. Do you daily consume candies?

□ Yes

□ No

5. Do you daily consume sugar-free chewing gums?

□ Yes

□ No

6. What do you drink when you are thirsty?

□ Water

□ Other beverage

**Part C: Addictive behaviors**

1. Do you smoke?

□ Yes

□ No

2. Do you consume cannabis?

□ Yes

□ No

3. Do you consume other drugs?

□ Yes

□ No

4. Do you consume alcohol?

□ Never

□ Occasionally

□ Daily

**Part D: Oral hygiene habits**

1. How many times do you brush a day?

□ Not every day

□ One time

□ Two times

□ Three times

2. What type of toothbrush do you use?

□ Manual

□ Electric

3. Where do you buy your toothpaste?

□ Pharmacy

□ Supermarket

4. Do you change your toothbrush regularly?

□ Yes, at least each three months

□ No

5. Do you use dental floss?

□ Yes

□ No

**Part E: Oral health-related behaviors**

1. Do you consider that your oral health is?

□ Good

□ Poor

2. Did you visit the dentist during the last year?

□ Yes

□ No

3. What was the reason for your last dental consultation?

□ Preventive

□ Curative

□ I do not know

4. Did you fail seeking dental care due to financial reasons?

□ Yes

□ No
